# Colonization history affects heating rates of invasive cane toads

**DOI:** 10.1038/s41598-020-69529-3

**Published:** 2020-07-28

**Authors:** Georgia K. Kosmala, Gregory P. Brown, Richard Shine

**Affiliations:** 1 School of Life and Environmental Sciences, The University of Sydney, Sydney, NSW 2006 Australia; 2 Department of Biological Sciences, Macquarie University, Sydney, NSW 2109 Australia

**Keywords:** Herpetology, Behavioural ecology

## Abstract

Amphibians in hot climates may be able to avoid high temperatures by controlling their rates of heating. In northern Australia, invasive cane toads (*Rhinella marina*) experience hot dry conditions in newly-colonized (western) sites but milder conditions in longer-occupied (eastern) sites. Under standardized conditions, toads from western sites heated less rapidly than did conspecifics from an eastern site. The availability of free water slowed heating rates of eastern but not western toads. Thus, the colonization of climatically extreme sites has been accompanied by a rapid shift in the toads’ ability to remain cool under hot conditions, even when free water is not available.

## Introduction

Widespread changes in climatic conditions have drawn attention to the mechanisms by which organisms deal with novel abiotic challenges such as extreme heat or aridity. One critical requirement is for organisms to maintain their body temperatures and hydric levels within narrow ranges relative to ambient conditions. Animals do so by a combination of behavioural, physiological and morphological traits^1^. For many types of animals, behavioural selection of appropriate microhabitats may be critical. For example, most adult anuran amphibians are nocturnally active, avoiding diurnal thermal conditions by remaining within moist and shaded retreat-sites by day^2,3^. If such sites are scarce, however, physiological control over the rate of heating may enable an anuran to keep its body temperature below lethal levels^4–6^. If the newly-invaded habitat is dry as well as hot, that physiological control over heating rate ideally should not depend upon access to free water. In moister regions, in contrast, increased rates of evaporative water loss may be an effective way to avoid lethal temperatures^4–6^.

The invasion of cane toads through tropical Australia provides an ideal model system with which to explore these ideas. The toads were released in 1935 along the well-watered coast of northeastern Australia, where temperatures are relatively mild and rainfall is plentiful year-round (e.g., Townsville, Queensland (Qld)—0 months per year with a combination of average maximum air temperature > 30 °C and rainfall < 25 mm^7^). The westwards invasion of this species has taken them into increasingly hotter and seasonally more arid regions to the northwest (number of consecutive months per year with mean maximum air temperature > 30 °C and < 25 mm rain = 3 months for Richmond, Qld; five for Middle Point, Northern Territory (NT); six for Kununurra, Western Australia (WA)). Toads in long-colonized areas of eastern Australia are relatively sedentary and frequently re-use the same diurnal refuges^2,3,8^ whereas toads at the western invasion front disperse whenever conditions allow it, sheltering by day under superficial vegetation and rarely re-using diurnal retreat-sites^9,10^. Many potential shelter sites in northwestern Australia reach lethally high temperatures^11^, forcing toads to abandon such shelters in search of water sources even if such dispersal is highly risky by day^12^. In summary, exposure to hot dry conditions may imperil survival of toads in northwestern Australia, but it is unlikely to be as serious a challenge in northeastern Australia. If toads are capable of physiological control over their rates of heating, we predict that individuals from cooler and moister eastern populations will heat up more rapidly than do conspecifics from the hotter and drier western populations. Also, we predict that access to free water will allow toads from eastern populations to heat up less rapidly (because they utilize water for evaporative cooling) whereas the western toads will not depend on free water to curtail rates of heating.

## Methods

**Figure 1:**
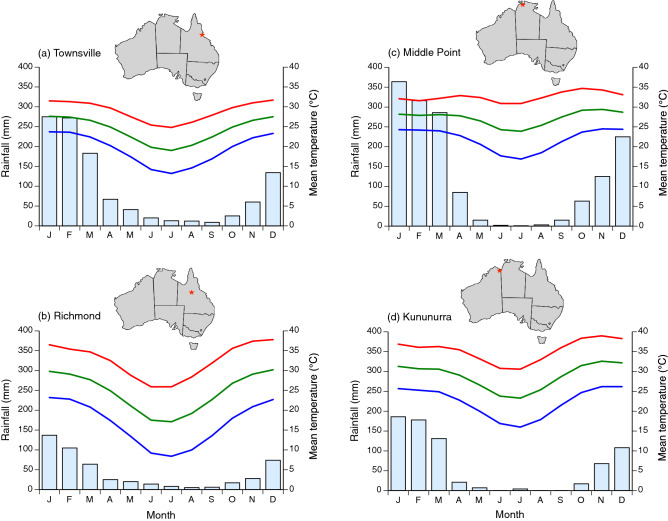
Mean climatic conditions in the four sites from which we collected cane toads (*Rhinella marina*) for use in laboratory trials. The red line connects mean monthly maximum air temperatures, the green line shows mean monthly air temperatures, and the blue line shows mean monthly minimum air temperatures. Histograms show mean monthly rainfall. Data from Australian Bureau of Meteorology^7^.

We hand-collected adult toads (*n* = 8 individuals per site) from four sites across the toads’ tropical range within Australia, from Townsville, Qld in the east (GPS coordinates: − 19.26, 146.82, 14 m altitude) to Richmond, Qld (− 20.73, 143.14, 218 m altitude), Middle Point, NT (− 12.56, 131.33, 12 m altitude) and Kununurra, WA (− 15.78, 128.74, 49 m altitude) in the west. That transect spans the toads’ 80-year invasion history. Although both temperatures and precipitation exhibit a general east–west cline, the greatest disparities in the duration of hot dry conditions per year lie between the easternmost site (Townsville) and the three other sites (Fig. 1). We recorded toad mass (after gently squeezing the animal in a standardized manner to induce it to empty its bladder) and snout-vent length (SVL) immediately before conducting the trials.

Toads were not fed for three days prior to experiments, to ensure they would not defecate during the experiment and minimize variability in mass due to stomach contents. Toads from all four populations were housed in a room kept at 18 °C, then moved concurrently to a temperature-controlled room set at 37 °C. All toads were in separate containers (ventilated plastic boxes of 1-L capacity), half of which had dry paper towel as substrate whereas the other half had 40 mL of water, enough to keep the ventral portion of the body moist but not the rest of the body.

We measured toad body temperatures at the beginning of the trial, and after 20 min and 40 min, using an infrared thermometer (Digitech QM7215) held ~ 10 cm from the toad’s dorsal surface. At the beginning and end of the experiment we measured internal temperatures with a cloacal probe (Digitech QM7215 with probe attachment), to check that our measurements of external body temperature offer robust estimates of internal temperature also. Cloacal temperatures were taken within 10 s of each toad’s removal from the container. After a trial, toads were kept at a temperature of 25 °C, allowed to fully hydrate and monitored for wellbeing during recovery. No adverse effects of the trials were evident.

We used mixed model repeated measures analysis to identify factors affecting body temperatures of cane toads during the 40-min heating trials. Sex and body mass were used as covariates in the analysis with climate at each collection site (# consecutive months per year with average maximum temperature > 30 °C and with < 25 mm rainfall (see above)) and wet/dry treatment as factors and time (0, 20, 40 min) as the repeated measure. Individual ID nested within population was used as a random effect. Our measure of climate characteristics at each site (0, 3, 5, or 6 hot dry months per year) was analysed as a continuous variable. However, for graphical purposes, our figures present data for each site depicted as a categorical variable (by location name). The data conformed to assumptions of normality and variance homogeneity and analyses were run using JMP Pro 14.0 (SAS Institute, Cary, NC).

### Ethical approval

All procedures were approved by the University of Sydney Animal Ethics Committee (Protocol #703), and all procedures conformed to Australian guidelines for the care and use of animals during research.

## Results

Mean body masses of toads were greater for females than for males (mean masses 177.7 vs. 129.4 g respectively; *F*_1,31_ = 9.42, *P* = 0.008) but did not differ significantly among sites (*F*_3,31_ = 2.81, *P* = 0.06; mean SVLs *F*_3,31_ = 2.61, *P* = 0.07; sex ratios Kununurra 2M, 5F; Middle Point 4M, 4F; Richmond 2M, 6F; Townsville 5M, 4F; mean body sizes ± SE = Kununurra 150.51 ± 17.19 g, 118.27 ± 3.71 mm SVL; Middle Point 174.08 ± 16.08 g, 117.89 ± 3.47 mm SVL; Richmond 184.89 ± 16.08 g, 122.39 ± 3.47 mm SVL; Townsville 126.03 ± 15.16 g, 109.49 ± 3.27 mm SVL). Body mass influenced the rate of heating [temperature at 20 min into the trials (but not at 0 or 40 min) and heating rate declined with toad mass (*r*^2^ = 0.20, *n* = 32, *P* < 0.01)].

**Figure 2:**
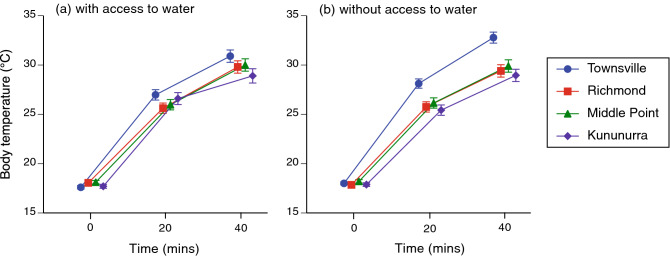
Body temperatures of cane toads (*Rhinella marina*) in laboratory trials in which the animals were transferred from cool to hot conditions. Symbols are displaced along the X-axis to avoid overlap. Toads were sourced from four locations with different climates; the fastest rates of heating were seen in toads from Townsville, the coolest and wettest site.

On average, toads heated from 17.9 to 30.2 °C over the course of the 40-min trials (Fig. 2). External body temperatures were highly correlated with simultaneously-measured internal temperatures on the same toads (*r*^2^ = 0.96, *F*_1,32_ = 1668.9, *P* < 0.0001; mean disparity < 0.01 °C; mean absolute disparity 0.40 °C).

**Figure 3:**
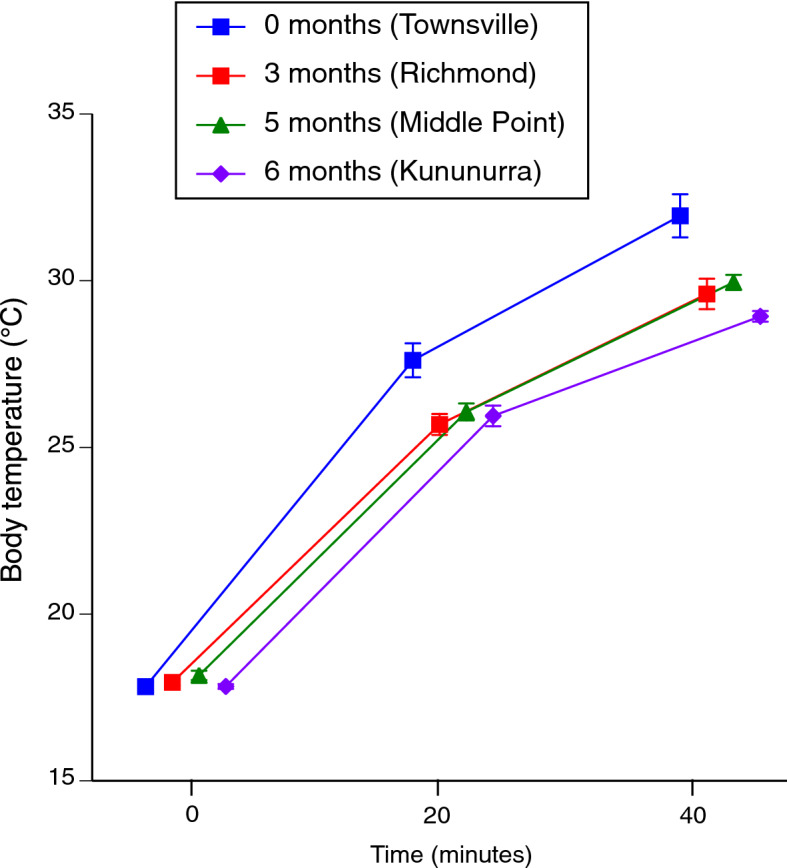
The interactive effect of climate (# consecutive months with average maximum air temperature > 30 °C and < 25 mm rainfall) and time (within trial) on body temperatures of cane toads during 40-min heating trials. Toads from the population that experiences the coolest and wettest conditions (Townsville, average of 0 consecutive hot dry months per year) heated more quickly than did toads from other sites (3–6 consecutive hot dry months per year). Data are pooled for both water treatment groups.

**Figure 4:**
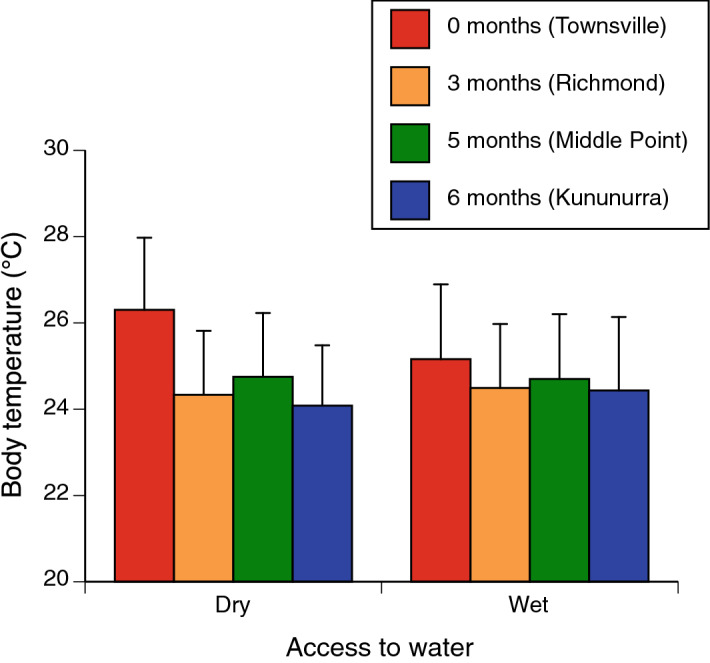
The interactive effect of climate at sites of collection (# consecutive months > 30 °C and < 25 mm rain) and presence of water during the trial, on body temperatures of cane toads during 40-min heating trials. When trials were conducted without access to water, toads from the population that experiences the coolest and wettest conditions (Townsville, 0 consecutive hot dry months per year) had higher average body temperatures than did toads from populations exposed to hotter drier climates. Data on toad temperatures are averaged across readings taken at 0, 20 and 40 min.

The statistical analysis revealed two significant two-way interactions involving climatic conditions of the sites from which toads were collected. The first interaction term indicated that although all toads commenced trials at the same temperatures, individuals from the population with the coolest moistest climate (Townsville) heated faster than did toads from other populations (climate × time interaction *P* < 0.015, Fig. 3). Second, the effect of water treatment on body temperature depended on the climate from which the toads originated. When access to water was removed, toads from the coolest moistest climate (Townsville) exhibited higher body temperatures than did conspecifics from hotter drier locations (climate × water interaction *P* < 0.02, Tables 1, 2, Fig. 4).

**Table 1:** Results of mixed model repeated measures analysis on factors affecting surface temperatures of cane toads. Sex and body mass were used as covariates in the analysis which focused on interactive effects of climate at each capture site (# consecutive months per year with average maximum air temperature > 30 °C and < 25 mm rainfall), treatment (presence or absence of water) and time (0, 20, 40 min). Individual ID nested within population was used as a random effect. Bold font indicates significant differences (*P* < 0.05).

Source	df	*F*	*P*
Sex	1, 26	3.36	0.0784
Body mass	1, 26	0.13	0.7222
Climate	1, 26	20.35	**0.0001**
Water	1, 26	0.89	0.3546
Time	1, 60	850.90	**< 0.0001**
Climate × water	1, 26	6.55	**0.0166**
Climate × time	1, 60	6.44	**0.0138**
Water × time	1, 60	0.16	0.6935
Climate × water × time	1, 60	0.60	0.4434

**Table 2:** Parameter estimates and standard errors from a mixed model repeated measures analysis on factors affecting surface temperatures of cane toads. Sex and body mass were used as covariates in the analysis which focused on interactive effects of climate at each capture site (# consecutive months per year with average maximum temperature > 30 °C and < 25 mm rainfall), treatment (presence or absence of water) and time (0, 20, 40 min). Individual ID nested within population was used as a random effect.

Term	Estimate	Std error
Intercept	19.568	0.453
Sex [F]	− 0.217	0.119
Body mass	− 0.001	0.003
Climate	− 0.203	0.045
Water [dry]	0.100	0.106
(Climate − 3.3125) × water [dry]	− 0.111	0.043
Time	0.306	0.010
(Climate − 3.3125) × (time-20)	− 0.012	0.005
Water [dry] × (time-20)	0.004	0.010
(Climate − 3.3125) × Water [dry] × (time-20)	− 0.003	0.005

## Discussion

The heating rates of toads largely conformed to our predictions. Toads from a population that experiences a relatively brief period of hot dry conditions each year (Townsville) heated faster than did conspecifics from locations that experienced hotter drier conditions for extended periods, and this disparity was greatest if the toads did not have access to free water (Figs. 2, 4). Those patterns are consistent with the hypothesis that anurans can exert some level of control over heating rates, and that this ability is most developed in animals from climates where hot and dry conditions imperil organismal viability. The disparity in heating rates between Townsville toads with versus without access to free water suggests that if water is available, these animals use it to increase evaporation rates from the skin surface and hence, slow down their rate of heating. Interestingly, however, no such disparity was evident in conspecifics from hotter drier climates (Figs. 2, 4). In the seasonally dry landscapes of northwestern Australia, water reserves are critical to toad survival^12,13^ and thus, toads in hot dry regions may benefit from an ability to curtail heating rates without expending water in the process.

The challenges of finding cool moist diurnal retreat sites may be greater for individuals at the expanding range edge of a biological invasion, than it is for philopatric individuals in long-established populations. Living in the same area for a long period allows an organism to learn the location of suitable shelter-sites, whereas a recently-arrived organism at an invasion front has no such opportunity. As a result, individuals that are at the forefront of an invasion (or are translocated) may be forced to utilize retreats that provide less-than-optimal thermal regimes. Radiotelemetric studies of cane toads have shown that individuals encountering novel habitats tend to shelter in more superficial (exposed) sites^14^, and hence exhibit higher body temperatures than do philopatric conspecifics^15^. That situation may exacerbate the thermal and hydric challenges that a colonizing toad encounters as the invasion front moves into hot dry climates (as in north-western Australia).

Although geographic variation in the seasonal duration of hot dry conditions across our collection sites was significantly correlated with patterns of variation in body temperatures during the heating experiment, our data are not sufficient to demonstrate causation. Other factors also vary among toads from the four sites that we studied, including physiology^19^, behavioural syndromes^20^ and morphology^21^. Thus, the geographic pattern that we found in rates of heating might be influenced by such characteristics, rather than (or as well as) by climatic variation. Future work could usefully attempt to tease apart the roles of such factors, by employing larger sample sizes and looking for other correlates of rates of heating. It would also be instructive to examine common-garden-raised offspring of field-collected toads, to tease apart the extent to which rates of heating are affected by phenotypically plastic responses to the conditions experienced during an individual’s life^20^.

Our data do not reveal the proximate mechanisms by which toads control rates of heating. Because the small size of the experimental enclosures gave minimal opportunities to manipulate heating rates by behavioural adjustments, morphological and/or physiological mechanisms are likely to be involved. Our measurements of skin thickness have revealed strong geographic heterogeneity in this trait, but with no close correspondence to the geographic variation in heating rates documented in the current paper^16^. Physiological mechanisms may be more important. For example, decreases in heartrate or shifts in blood flow (away from peripheral vessels) might reduce rates of heat uptake^17^. Future work could usefully explore the mechanisms by which toads can control their own rates of heating; and also, whether the geographic divergence in heating rate, and in the effect of water availability on heating rate, are driven by adaptation (via genetic and/or epigenetic changes) or phenotypic plasticity (induced by exposure to harsh conditions). Despite the brief timespan of the toads’ colonization of northwestern Australia (decades only), a complex mix of heritability and plasticity underlie other geographically-divergent traits in this species^18–21^. Although mechanisms remain unknown, the capacity of cane toads in hot dry climates to reduce heating rates, even if they have no access to water, may well have contributed to the remarkable success of this invasive anuran in the harsh climatic conditions of the Australian outback.

## Data Availability

Data are available at 10.5061/dryad.cvdncjt24.
